# On stability and associative recall of memories in attractor neural networks

**DOI:** 10.1371/journal.pone.0238054

**Published:** 2020-09-17

**Authors:** Suchitra Sampath, Vipin Srivastava

**Affiliations:** 1 Centre for Neural and Cognitive Sciences, University of Hyderabad, Hyderabad, Telangana, India; 2 School of Physics, University of Hyderabad, Hyderabad, Telangana, India; Queen Mary University of London, UNITED KINGDOM

## Abstract

Attractor neural networks such as the Hopfield model can be used to model associative memory. An efficient associative memory should be able to store a large number of patterns which must all be stable. We study in detail the meaning and definition of stability of network states. We reexamine the meanings of retrieval, recognition and recall and assign precise mathematical meanings to each of these terms. We also examine the relation between them and how they relate to memory capacity of the network. We have shown earlier in this journal that orthogonalization scheme provides an effective way of overcoming catastrophic interference that limits the memory capacity of the Hopfield model. It is not immediately apparent whether the improvement made by orthgonalization affects the processes of retrieval, recognition and recall equally. We show that this influence occurs to different degrees and hence affects the relations between them. We then show that the conditions for pattern stability can be split into a necessary condition (recognition) and a sufficient one (recall). We interpret in cognitive terms the information being stored in the Hopfield model and also after it is orthogonalized. We also study the alterations in the network dynamics of the Hopfield network upon the introduction of orthogonalization, and their effects on the efficiency of the network as an associative memory.

## 1 Introduction

Associativity is a fundamental feature of learning and memory. When some information is learnt or memorized, it can be recalled not just when the same information is encountered again, but also by similar or partial information. The brain thus forms *associations* between the various information it learns and memorizes with those it encounters externally. This kind of associative memory can be modeled mathematically using some ideas from physics and mathematics which can be adapted to neuronal networks [1–6]. Such models of networks can help us gain insights into the mechanisms underlying learning and memory.

An attractor neural network (ANN) can be used to model associative memory [7]. Information is presented to the network as vectors, which can be sequences of numbers, usually ±1’s forming a variety of patterns [1, 8, 9]. A set of patterns learnt by the network should form fixed points in the dynamics of the network and act as ‘attractors’. An attractor is surrounded by a region containing a set of patterns associated with it. These regions are referred to as *basins of attraction* of the attractors [7, 10]. On presenting a pattern within the basin of attraction of an attractor, the network can recover the pattern that has been learnt and identifies the attractor. The network is thus capable of ‘error correction’, as the learnt patterns can be recovered even on presenting different or erroneous forms of these patterns. The network is also capable of classifying or categorizing information, as separating patterns into basins of attraction amounts to categorization [10].

It is crucial for patterns inscribed in the network to be stable in order for the system to be functional as memory. In other words, if we wish to store some information in a network, then the information must become a stable fixed point in the network dynamics. An additional requirement for the network to be effective as an associative memory is that the information must not only be a stable fixed point, but must also be an attractor. We will explain shortly the reason for making this distinction. The main aim of this paper is to explain the meaning and importance of stability of the state of a network that represents a memory and how it is affected by the network dynamics. The terms retrieval, recognition and recall are often used interchangeably to refer to process of associativity or recovery of learnt patterns. However, in the context of stability, there are subtle differences between them, which we will underline here.

It is well known that the quality of associativity in Hopfield network deteriorates rapidly as information piles up beyond a (rather low) threshold. This arises due to catastrophic interference between memories resulting from correlations between the learnt patterns. To circumvent this problem, an orthogonalization scheme was proposed [6, 11, 12]. It proved to be successful in enhancing the memory capacity and gave new insight into cognitive learning and memory. However, it is not inherently clear whether orthogonalization can affect the network dynamics to different extents for the processes of retrieval, recognition and recall. Hence it is necessary to define these terms clearly and study how their relation to each other is modified by orthogonalization and what these modifications mean for pattern stability. We do so in this paper, by extending our study reported in [5] to address deeper questions about how the stability of memories, and consequently associativity are affected by orthogonalization.

For completeness and ease of comparison, we first recapitulate briefly the Hopfield network in sec. 2. In sec. 2.1, we discuss the various meanings assigned to the terms retrieval, recognition and recall in various fields and present our precise definitions of these terms. Following this, we state the requirements for pattern stability (sec. 2.2) before exploring in detail the basins of attraction of various patterns in the network in sec. 2.3. We then study the dynamics of the network and the energy landscape in sec. 2.4. Finally, we calculate the memory capacity of the network in sec. 2.5. We then recapitulate in sec. 3 how orthogonalization is introduced in the Hopfield framework, and discuss how the basins of attraction are modified in this model(sec. 3.1). In subsequent subsections, we examine the energy landscape, network dynamics and memory capacity of the network with orthogonalization. A comparison of the effect of correlations between the patterns on the efficiency of the Hopfield model and our modified model is presented in sec. 4. After presenting these results, we discuss their relevance to the network as an associative memory and also to cognition.

## 2 The Hopfield model with Hebbian learning

The Hopfield model [1] is a network of *N* ‘neurons’ connected with each other through ‘synapses’. A neuron can take values +1 or -1, depending on whether it is firing (active) or not (inactive). Each neuron is connected to every neuron in the network except itself. Synapses are characterized by their ‘weights’ or ‘efficacies’, which can increase due to ‘potentiation’, or decrease due to ‘depression’ by collective activities of the neurons connected to it through the synapses. Information is presented to the network in the form of N-dimensional vectors whose components represent the activities on each neuron. These patterns are believed to be stored or inscribed in the network by cumulatively modifying the weights following the prescription proposed by Donald Hebb [13]. The Hebbian learning rule can be represented mathematically as,

$$\begin{array}{c} J_{ij}=\frac{1}{N}\sum_{\mu =1}^{p}\sum_{{}_{i\neq j}^{i,j=1}}^{N}\xi_{i}^{\left(\mu \right)}\xi_{j}^{\left(\mu \right)}, \end{array}$$ (1)

where *J*_*ij*_ is the weight on the synapse connecting the pre-synaptic neuron *j* to the post-synaptic neuron *i* after *p* patterns have been inscribed in the network. $\xi_{i}^{\left(\mu \right)}$ and $\xi_{j}^{\left(\mu \right)}$ give the activities on the *i*^*th*^ and *j*^*th*^ neurons in a pattern *μ*, while $\frac{1}{N}$ is the normalizing factor. This learning rule is Hebbian in that when two neurons fire simultaneously, the synapse connecting them is strengthened while a synapse is depressed if only one of the neurons connected by it is active. However, a synapse in the model is strengthened even when neither of the neurons connected by it is active. This appears to be biologically implausible. Moreover, the weights are assumed to be symmetric for a mathematical reason, i.e., *J*_*ij*_ = *J*_*ji*_, which also does not seem biologically realistic. Despite such apparent shortcomings, the network has proved to be a useful tool to understand learning and memory.

The learning rule, after Eq (1) ensures that the inscribed patterns ***ξ*^(*μ*)^**’s minimize an energy function, or Hamiltonian, given by,

H=−12∑i≠ji,j=1NJijξi(μ)ξj(μ). (2)

The inscribed patterns are thus fixed points in the network dynamics and act as minima in an energy landscape.

### 2.1 Retrieval, recognition and recall

The discussions that follow necessitate that we examine closely the meanings of the terms *retrieval, recognition* and *recall*, which are otherwise often used interchangeably in physics and neuroscience literature. The cognitive-psychology literature tends to be relatively more discrete but it is difficult to always find exact correspondence in meanings of the terms there and these three terms. Further, we need to attribute mathematical representations to the cognitive terms.

If an information already in the memory store is presented to the network and the network is able to associate it exactly and spontaneously with the stored information, then we say that the network has *retrieved* that information. In our scheme of things *exact mapping* and *spontaneity* are central to the process of *retrieval*. However, in the course of mapping the network may not always reproduce the learnt information accurately right away, *i.e.* in the first attempt, instead it may first produce something very similar, which may subsequently lead to the reproduction of the exact information that is there in the store in the next one or a few attempts. We distinguish it from *retrieval* and say that the network has *recognized* the presented information as one in the memory store. Note that the process of *recognition* has two parts to it, which we will identify after explaining our meaning of *recal*.

If we come across something that is similar to, but not exactly the same as, something that we have in the memory store, yet the network is able to connect it exactly with the latter, either instantaneously or in a few attempts, then we say that the network is able to *recal*. *Recal* is like *pattern completion or error correction*. (Pattern completion or error correction refers to the process of recovering a correct learnt memory when partial or erroneous information is presented to the network. [14–17].) The input information is different from an inscribed information but it leads to *recollection* of the inscribed one.

Now, we can see that in the first part of *recognition* the network reproduces something that reminds us of a stored information, or, in other words, indicates that what has been reproduced is quite *familiar*, in the same sense as in the psychology literature [18–23]. The second part is clearly the same as *recal*. Thus, like in cognitive psychology literature, in our case also *recognition* corresponds to the situation where a presented information first produces the impression of being *familiar* and then *recalls* the exact stored information.

We will come across another situation in which when an inscribed information (or pattern) is presented to the network for association, it is neither *retrieved* nor *recognized*, but it gets associated with an unknown pattern, which was not inscribed but is similar to the inscribed pattern. We will elaborate on it later on after introducing the concept of stability of inscribed patterns or information.

It is important to note that our network is capable of *associative recall* as long as each of the inscribed information or patterns has a set of patterns associated with it, as the network dynamics leads us through a set of similar but unfamiliar patterns before reaching a memorized pattern.

We will now see how the above observations fall in place in the following mathematically precise definitions of *retrieval*, *recognition* and *recal* in mathematical terms. A pattern ***ξ*^(*ν*)^** is considered recovered or stable if,

$$\begin{array}{c} sgn(h_{i}^{\left(\nu \right)})=sgn(\xi_{i}^{\left(\nu \right)}),\;\text{for}\;\text{all}\;i’\text{s}, \end{array}$$ (3)

where,

hi(ν)=∑i≠jj=1NJijξj(ν), (4)

gives a local field potential or post-synaptic potential on neuron *i*, which is created collectively by the activities on the remaining *N* − 1 neurons in pattern *ν* and projecting onto *i* via *j_ij_*’s. The *sgn* on either side of the equation checks whether the signs of ***h*^(*ν*)^** and ***ξ*^(*ν*)^** on each neuron *i* match. Each instance of these equations is referred to as an *iteration*.

In the forthcoming analysis we will use ***ξ*^(*ν*)^** as the generic notation for the inscribed patterns. One of the inscribed patterns, or a pattern that is similar to it, will be arbitrarily picked up to test how it is recovered when it is subjected to the protocol stipulated in eqs. (3) and (4). This test pattern will be designated as ***ξ*^(*t*)^**. The test pattern ***ξ*^(*t*)^** will be subjected to (not more than) a specified maximum number of iterations in the retrieval prescription of Eq (3), and checked for recovery with perfect (or 100%) accuracy, i.e. without a single mismatch. Now,

- if ***ξ*^(*t*)^** = ***ξ*^(*ν*)^**, that is, the test pattern is chosen to be identical to an inscribed pattern ***ξ*^(*ν*)^**, and if on presentation of ***ξ*^(*t*)^** to Eqs (3) and (4), ***ξ*^(*ν*)^** is recovered instantaneously, i.e., in a single iteration, then ***ξ*^(*ν*)^** is said to be *retrieved*; and again
- if ***ξ*^(*t*)^** = ***ξ*^(*ν*)^**, but it takes more than one iteration to converge to ***ξ*^(*ν*)^**, then ***ξ*^(*ν*)^** is said to be *recognized*. That is,***ξ*^(*t*)^** settles down to ***ξ*^(*ν*)^** within a certain specified number of iterations and stays there even when subjected to further iterations; but
- if ***ξ*^(*t*)^** is *not* identical to ***ξ*^(*ν*)^** but somewhat similar to it (i.e. there are some mismatches between ***ξ*^(*t*)^** and ***ξ*^(*ν*)^**), yet ***ξ*^(*t*)^** converges to ***ξ*^(*ν*)^** within a specified number of iterations, then ***ξ*^(*ν*)^** is said to be *recalled*.

**Fig 1:**
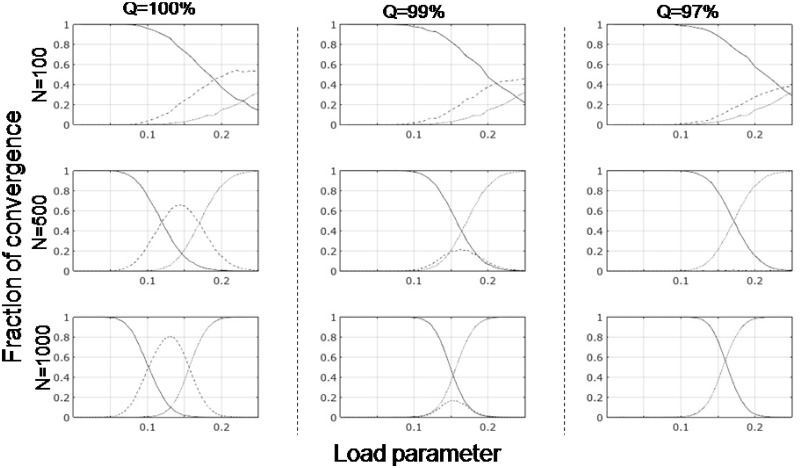
Quality of convergence as a function of load parameter. Plot showing where the inscribed patterns in the Hopfield network converge for various values of *N* (*N* = 100, 500, 1000), for different qualities of convergence (*Q* = 100%, 99%, 97%). A pattern can converge either to itself (solid line) or to a different pattern (dashed line), depending on the error tolerance for that value of *Q*. The thin dotted line indicates lack of convergence even after 10 iterations. For *Q* = 100%, the error tolerance is 0%, and only a perfect match is treated as converging to ‘itself’. For *Q* = 99% and 97%, the error tolerances are 1% and 3% respectively, which means that convergence to a nearby pattern is acceptable in such calculations. The plots show data from 50 different sets of patterns.

The reason we make a distinction between retrieval and recognition is that in the latter case, a test pattern may visit an inscribed pattern in the course of settling down to an attractor which may not be the same pattern as the test/inscribed pattern. Refer to the SI for an example of this situation. The crucial condition for a pattern in the network to be an attractor is that it should converge to itself with perfect accuracy i.e, there should be a 100% match between that pattern and the pattern to which it converges eventually. In the following, we refer more often to *recognition* and *recall* than *retrieval*, as convergence does not happen so often in one iteration specially due to the strict condition of 100% match. The reason behind our choice of this strict condition of perfect accuracy between the presented and recovered patterns will become clear in the next section, while in most cases an accuracy of ≥97% is deemed acceptable [7]. The link between convergence quality and accuracy is shown in Fig 1, while their cognitive implications are discussed later.

### 2.2 Criteria for memory stability

We now turn to working out the stability criteria for memories stored in the network, as memory stability is crucial to the efficient functioning of the network as an associative memory.

As we have seen, the condition in Eq (3) cannot be used as the sole criterion for pattern stability, as it does not guarantee convergence of a test pattern to itself. A test pattern may lead to an inscribed pattern being recovered but may eventually converge to a different pattern. A mismatch (or error) in one iteration can in principle lead to increase in mismatches on subsequent iterations, resulting in an ‘avalanche’ of errors [8]. So, the necessary condition for pattern stability should be *recognition*.

To understand how a stable pattern can become unstable we analyse the condition (3) in terms of signal and noise. For this we rewrite the equation in terms of a stabilization parameter $s_{i}^{\left(\nu \right)}$ [7, 9] given by,

$$\begin{array}{c} s_{i}^{\left(\nu \right)}=h_{i}^{\left(\nu \right)}\xi_{i}^{\left(\nu \right)}, \end{array}$$ (5)

and then plug in *J*_*ij*_ from (1) into (4) to get,

$$\begin{array}{c} \begin{array}{ccc} h_{i}^{\left(\nu \right)} & =\sum_{{}_{j\neq i}^{j=1}}^{N}J_{ij}\xi_{j}^{\left(\nu \right)} & =\frac{1}{N}\sum_{{}_{j\neq i}^{j=1}}^{N}\sum_{\mu =1}^{p}\xi_{i}^{\left(\mu \right)}\xi_{j}^{\left(\mu \right)}\xi_{j}^{\left(\nu \right)}. \end{array} \end{array}$$ (6)

Separating the $\xi_{i}^{\left(\nu \right)}$ terms from the rest on the right hand side of the equation, we get,

$$\begin{array}{c} h_{i}^{\left(\nu \right)}=\xi_{i}^{\left(\nu \right)}+\frac{1}{N}\sum_{{}_{\mu \neq \nu }^{\mu =1}}^{p}\sum_{{}_{j\neq i}^{j=1}}^{N}(\xi_{i}^{\left(\mu \right)}\xi_{j}^{\left(\mu \right)}\xi_{j}^{\left(\nu \right)}). \end{array}$$ (7)

Now, the stabilization parameter is,

$$\begin{array}{c} s_{i}^{\left(\nu \right)}=1+\frac{1}{N}\begin{array}{c} \sum_{{}_{\mu \neq \nu }^{\mu =1}}^{p}\sum_{{}_{j\neq i}^{j=1}}^{N}(\xi_{i}^{\left(\mu \right)}\xi_{j}^{\left(\mu \right)}\xi_{j}^{\left(\nu \right)})\xi_{i}^{\left(\nu \right)}. \end{array} \end{array}$$ (8)

This expresses the stabilization parameter in terms of a signal, the inscribed pattern ***ξ*^(*ν*)^**, and a noise term due to correlations between the pattern ***ξ*^(*ν*)^** and all the other patterns. This noise arising from the overlaps between the patterns is referred to as *slow noise*. Note that this noise term can be negative with its chances of becoming negative increasing with *p*. A pattern is stable as long as it has a positive stabilization parameter. As we have discussed earlier, as long as $s_{i}^{\left(\nu \right)}>0$ for all *i*’s, ***ξ*^(*ν*)^** will be retrieved (or recognized, as the case may be) perfectly, or recalled accurately if the presented pattern ***ξ*^(*t*)^** deviates from ***ξ*^(*ν*)^**. Thus, a pattern is stable as long as the signal due to the pattern stands out against the noise.

For a pattern ***ξ*^(*ν*)^** to be considered stable, it must satisfy the following criteria (along the lines of Bar-Yam [10], also refer to [8] and [9] for other discussions pertaining to the conditions for pattern stability):

1. It should be recognized, i.e., when presented, it should be recovered with 100% accuracy within a small number of iterations; and
2. It should be an attractor in the network dynamics and have a basin of attraction around itself. That is, the network can recall ***ξ*^(*ν*)^** when presented with any of the patterns within its basin of attraction, all of which converge to ***ξ*^(*ν*)^**.
3. It should have a positive stabilization parameter $s_{i}^{\left(\nu \right)}>0$ for all *i*’s

The first two conditions are connected with each other because we have found computationally that if an inscribed pattern, when presented for recognition, does not converge to itself with 100% accuracy, then it will not be an attractor, that is, it won’t have a basin of attraction around it. It may converge to an unknown pattern, and this new pattern will be an attractor while the inscribed pattern would lie in its basin of attraction; besides, the new pattern would converge to itself when presented for recovery. In this manner the new unknown pattern would be a stable state of the system. The converse is true in a wider sense in that an attractor (with a basin around it), whether or not an inscribed pattern, will converge to itself when subjected to the iterative procedure of recovery. Thus while the condition 1 may be treated as a necessary condition for a pattern to be stable, whether or not it is inscribed, the condition 2 will be the sufficient condition for stability. Note that condition 3 is satisfied as long as condition 1 is satisfied.

We will revisit the above conditions for the stability of a pattern and study their applicability to the model with orthogonalization. The modified model will present a situation where the above conditions will be challenged—a scenario will arise where patterns are recognized (or sometimes retrieved) accurately but will lack basins of attraction. That is, initially the patterns all form stable attractors and eventually become stable non-attractors depending on the amount of information stored in the network. (Stable non-attractors refer to fixed points that are stable, but not attractors.) We will hence claim that while the necessary condition will be met but not the sufficient condition, yet the patterns will comprise stable states of the network. This will compel us to reexamine the meaning of basins of attraction of size zero.

Since basin of attraction has emerged as something central to the stability of patterns, we will explore in detail the network dynamics of the Hopfield network before investigating how the network dynamics and the basins of attraction derived from them change when orthogonalization is introduced.

### 2.3 Basins of attraction

We shall first discuss briefly the shape of basins of attraction and how they are calculated (following [10]) before studying the network dynamics and calculating the memory capacity of the network.

Imagine an *N*+ 1 dimensional space—*N* space and 1 energy dimension—in which a point represents the location of an *N*-dimensional vector ***ξ*^(*ν*)^** (which is a configuration of pattern of ±1’s) and its energy. We can visualize an energy landscape in this ‘configuration space’ with hills and valleys. The inscribed patterns are expected to be at the bottoms of the valleys. In fact when a pattern is inscribed in the Hopfield network, it should create a valley for itself and sit at the bottom because it should minimize the total energy of the system. A whole lot of patterns in its neighbourhood would form the valley and satisfy the condition that if any of these is presented for recall following Eq (3), it would converge to the inscribed pattern at the bottom of the valley.

For mathematical reasons (which may not be important to be elaborated here), the *N*+ 1 dimensional configuration space should be finite, but large enough to accommodate 2^*N*^ patterns. So, as new patterns are added (starting from a small number, say less than 10% or so of *N* the topography of the energy landscape continuously changes to make room for new patterns. In the process the energy minima shift around, the basins reduce in size and also possibly become shallower. As a result some of the old minima may get replaced by new minima, i.e some of the inscribed patterns may cease to be at the bottom of their valleys; the minima now correspond to new patterns that may not be among the inscribed ones while the inscribed patterns may occupy off-minima positions in the basins.

The extent of the basin around a pattern is generally expressed in terms of an average radius around the bottom [10], which in the Hopfield network can be expressed in terms of Hamming distance that measures the number of mismatches between two patterns. However, the average value can be misleading, as the basin of attraction of a stable pattern refers to the entire structure around the minimum corresponding to the pattern and may seldom be uniform in every direction. It is thus more appropriate to represent a basin of attraction by a set of Hamming distances, rather than a single averaged value. We shall later justify this way of representation. Each Hamming distance gives the maximum difference between a test pattern ***ξ*^(*t*)^** and a pattern that lies at the bottom of a basin where the test pattern is the farthest from the latter in a particular direction but still converges to it.

The Table 1 shows that the Hamming distances constituting a basin of attraction can vary greatly. A basin is thus anisotropic and may extend more in some directions than in others. A stable pattern may have ‘0’ values for one or a few sample(s) within its basin, while an unstable pattern will have only 0’s within its basin, as unstable patterns do not converge to themselves. The unstable patterns may converge to other patterns, the new attractors, which have non-zero basins of attraction while the unstable inscribed patterns lack basins of their own.

**Table 1:** Table showing the basins of attraction of certain attractors corresponding to various inscribed patterns (*ξ*^(*ν*)^’s) for different values of *p* for *N* = 100. *ξ*^(*ν*′)^ represents the (new) attractor to which an unstable *ξ*^(*ν*)^ converges. The basin of attraction of a stable pattern can include a Hamming distance of value zero: 0 can be one amongst the various numbers representing the basin. The basin of attraction of *ξ*^(4)^ when *p* = 12 provides an example of this. In addition, we can see the differences in basins of attraction even between stable patterns like in *ξ*^(1)^ and *ξ*^(4)^ for the same value of *p*. Also note the anisotropy in the spread of the Hamming distances in the case of *ξ*^(4)^. The basins of attraction of the stable patterns shrink in most directions as *p* increases, as seen for *p* = 14 and *p* = 16. The new attractors *ξ*^(*ν*′)^ have non-zero basins of attraction.

	*ξ*^(*ν*)^	Hamming distances forming the basin of attraction of *ξ*^(*ν*)^									
*p* = 10	***ξ*^(1)^**	31	29	37	35	40	24	30	34	41	31
	***ξ*^(7)^**	44	39	48	39	39	32	40	36	35	37
*p* = 12	***ξ*^(1)^**	32	36	32	44	34	34	35	32	34	37
	***ξ*^(4)^**	0	4	6	7	3	6	6	8	1	19
	***ξ*^(7)^**	0	0	0	0	0	0	0	0	0	0
	***ξ*^(7′)^**	31	32	39	35	40	26	30	47	37	37
*p* = 14	***ξ*^(1)^**	26	31	36	41	37	22	30	31	31	39
	***ξ*^(7)^**	0	0	0	0	0	0	0	0	0	0
	***ξ*^(7′)^**	5	16	8	22	3	7	6	10	6	6
*p* = 16	***ξ*^(1)^**	31	21	8	27	28	35	27	33	17	28
	***ξ*^(7)^**	0	0	0	0	0	0	0	0	0	0
	***ξ*^(7′)^**	13	6	10	7	7	23	8	17	18	7

In brief, a basin is demarcated as follows. Starting from the pattern ***ξ*^(*ν*)^**, we flip its elements in a random sequence (called a ‘sample’) and check for convergence to ***ξ*^(*ν*)^** at each step. The process is stopped once the test for convergence fails. Note that the systematic flipping of spins is akin to raising the temperature and acts as a second source of noise, referred to as *fast noise*. The procedure is then repeated with different random sequences to obtain the basin of attraction of ***ξ*^(*ν*)^**. The entire process is repeated with a different set of inscribed patterns for the same range of *p*. A detailed explanation of the procedure is given in the SI.

### 2.4 Network dynamics and energy landscape

The Hebbian learning rule (1) ensures in principle that the ***ξ*^(*ν*)^**’s are in the memory store by minimizing the total energy (2) and by forming attractors. However the network dynamics experiences constraints as more and more information piles up; i.e. with increasing *p*. This can be examined by doing energy calculations (following [24]). The energy *E*(***ξ*^(*μ*)^**) corresponding to a pattern ***ξ*^(*μ*)^** (expressed as a row vector) is given by,

$$\begin{array}{c} E(\xi^{\left(\mu \right)})=-\frac{1}{2}\xi^{\left(\mu \right)}J{\xi^{\left(\mu \right)}}^{T}, \end{array}$$ (9)

where *J* represents the synaptic weights matrix and *T* represents transpose of a vector ***ξ*^(*μ*)^** which changes a column vector to a row vector and vice-versa. In this notation, after *p* patterns have been inscribed in the network, the weight matrix is given by,

$$\begin{array}{c} J=\sum_{\mu =1}^{p}{\xi^{\left(\mu \right)}}^{T}\xi^{\left(\mu \right)}-pI. \end{array}$$ (10)

where *I* represents the *N* × *N* identity matrix. The subtraction of identity matrix *I* helps in removing self-connections from the weights matrix.

From Eqs (5) and (9), we can see that,

$$\begin{array}{c} E(\xi^{\left(\mu \right)})=-\frac{1}{2}s^{\left(\mu \right)}, \end{array}$$ (11)

where $s^{\left(\mu \right)}=\sum_{i}s_{i}^{\left(\mu \right)}$. The Eq (8) shows that the stabilization parameter will be positive and large for *p* ≪ *N*, accordingly Eq (11) shows that the inscribed patterns become energy minima for those values of *p*. The average energy of the inscribed patterns and the attractors are scattered about a mean, typically *N*/2, as can be seen from Table 2. Although there is a change in the radii of the basins of attraction with variation in *p*, the energies of the attractors remain distributed around the same mean. This distribution is retained across networks of different sizes.

**Table 2:** Table showing the mean, or average energy of patterns, $E_{avg}^{\left(\mu \right)}$, and that of the attractors, $E_{avg}^{\left(\mu^{\prime }\right)}$, for a particular value of *p* for various *N*. The energies of the patterns are distributed around a mean, which is typically around *N*/2. Even as *p* increases, the energies of the patterns are still scattered around *N*/2.

*p*	Average Energy	*N* = 100	*N* = 200	*N* = 500	*N* = 700	*N* = 1000
0.10*N*	$E_{avg}^{\left(\mu \right)}$	−48.58	−100.18	−249.49	−349.63	−500.66
	$E_{avg}^{\left(\mu^{\prime }\right)}$	−48.58	−100.18	−249.49	−350.15	−501.14
0.14*N*	$E_{avg}^{\left(\mu \right)}$	−49.06	−98.62	−249.83	−349.55	−503.45
	$E_{avg}^{\left(\mu^{\prime }\right)}$	−49.06	−98.66	−251.70	−350.43	−504.46
0.16*N*	$E_{avg}^{\left(\mu \right)}$	−49.36	−102.24	−248.38	−350.95	−501.30
	$E_{avg}^{\left(\mu^{\prime }\right)}$	−49.62	−102.52	−250.34	−353.21	−503.94
0.20*N*	$E_{avg}^{\left(\mu \right)}$	−48.52	−102.76	−247.82	−352.95	−499.95
	$E_{avg}^{\left(\mu^{\prime }\right)}$	−49.00	−103.98	−249.83	−360.21	−504.42

When a new pattern comes to be stored in the network, the *J*_*ij*_’s are altered and the entire energy landscape is modified to accommodate this new pattern and its basin of attraction. In this process, the basins of attraction of the previously stored patterns may undergo large scale modifications; they may extend more in some directions and shrink in others and the energy minima may shift along with the possibility of becoming shallow. This results in the basins of attraction becoming more and more anisotropic, and the energy terrain becoming more and more tortuous. The addition of new patterns also causes an increase in the soft noise in the system. This building up of noise causes further reduction in the sizes of the basins of attraction, as the basins interfere with each other [25].

The imprinted patterns overlap not just with the patterns neighbouring them, but in different degrees with all the other patterns as we can see from Eq (7). This is because the network is fully connected. Thus, the addition of new patterns adds to cross talks and to the noise. As the noise increases, some of the inscribed patterns may no longer be recognized perfectly. That is, the noise becomes so high that the stabilization parameters (5) are no longer positive for all the neurons in these patterns: the noise *destabilizes* some neurons in the patterns which were previously stable, resulting in mismatches in the signs between the inscribed and retrieved patterns. The patterns thus become unstable now, as stability requires the signs on all neurons to match; and their basins of attraction vanish, or reduce to zero.

**Fig 2:**
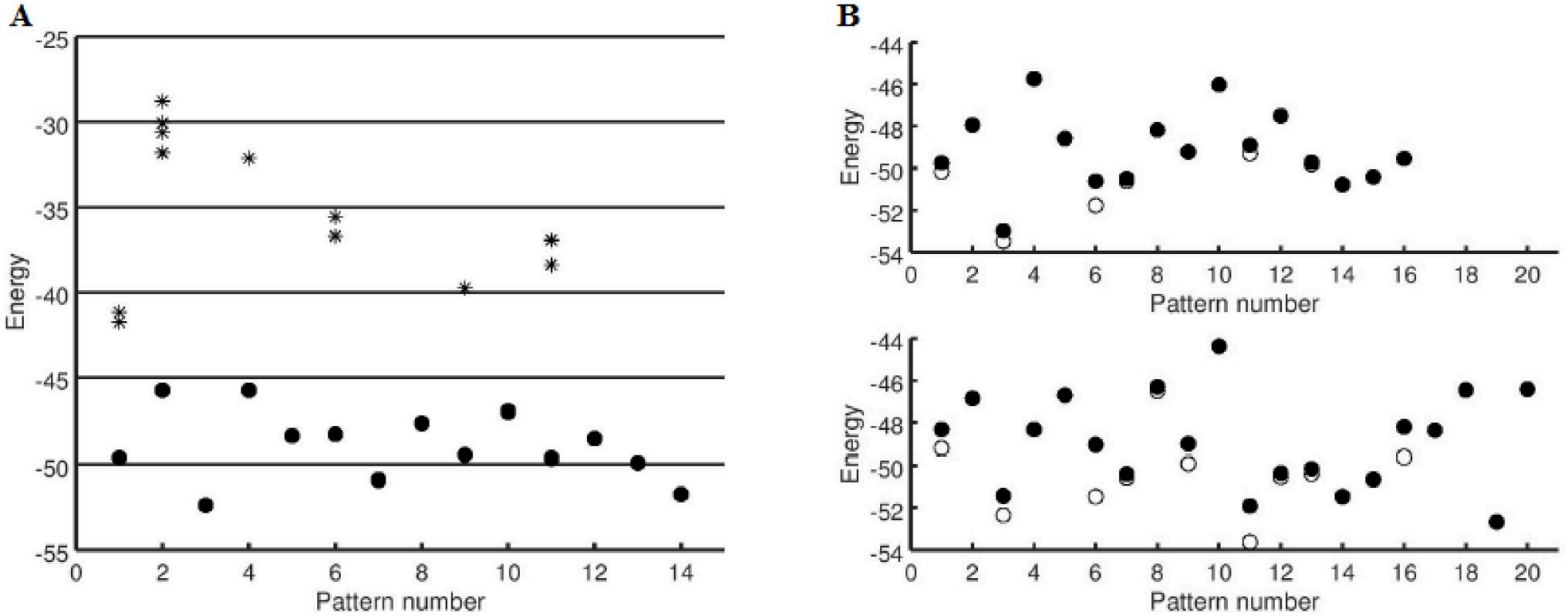
Energies of various classes of attractors. Plot showing the energies for the inscribed patterns (•) and the attractors(∘) for **(A)** *p* = 14 and **(B)** at *p* = 16 and 20 for *N* = 100. At *p* = 14, for stable patterns, the attractor corresponds to the inscribed pattern, indicated by the • and ∘ coinciding. For an unstable inscribed patterns (for instance, ***ξ*^(7)^)**, the attractor is at a slightly lower energy than the inscribed pattern but very close to it. (The difference between the attractors in the plot is not very distinct at this resolution). The *’s represent various symmetric mixture states with 3, 5 and 7 components, and are plotted against the inscribed pattern with which they have maximum overlap. These mixture states have much higher energy than any of the inscribed patterns. From Fig. **(B)**, we can see that even at such high values of *p*, there are still stable patterns, but with increase in *p*, the number of unstable patterns goes up. As *p* increases from 16 to 20, the attractors pertaining to unstable patterns move further away from the corresponding inscribed patterns.

To sum up, initially, all the patterns inscribed in the network are stable. The attractors corresponding to stable patterns are characterized by deep basins of attraction with energy at the bottom being the minimum of the Hamiltonian (2). As the inscribed patterns reorganize themselves and make room to accommodate new patterns, some of the attractors move away from the positions where the corresponding inscribed patterns were located, rendering those inscribed patterns unstable. These new attractors, which are initially close to the inscribed patterns, move further and further away when more patterns come to be stored in the network. This is depicted systematically in Fig 2**(A)** and 2**(B)** as a function of increasing *p*.

The inscribed patterns are not the only attractors in the energy landscape. When patterns are inscribed in the memory, their mirror images also form minima. Additionally, there are other attractors referred to as *spurious minima* or false memories, as they form attractors, but do not coincide with any of the inscribed patterns. They are typically formed from combinations of the inscribed patterns. Some such states are shown in Fig 2**(A)**. Refer to the SI for examples of spurious states and the basins of attraction of various classes of attractors in the energy landscape.

### 2.5 Memory capacity

A learnt pattern should minimize the Hamiltonian for it to be retained in the memory. But we have seen that as the memories pile up some of the incoming patterns that are also inscribed may not minimize the total energy, instead some other nearby patterns may do so. These energy minima correspond to patterns that have not been inscribed or learnt. So these ‘unknown’ patterns cannot be characterized or counted as memories. We, therefore, need to redefine the memory capacity.

In the background of the finer distinction we have made between retrieval, recognition and recall it is obvious that memory capacity should comprise only those inscribed patterns that can be recognized. This is because they form minima in the energy landscape, and also have basins of attraction around them. The latter ensures that they can be recalled in case a nearby pattern happens to be encountered. The inscribed patterns that do not minimize the energy can neither be recognized nor recalled because they do not have basins of attraction of their own.

Our analysis thus underlines the significance of recognition/recall with 100% overlap with a learnt pattern. Our estimate of memory capacity will therefore be less than *p*/*N* = 0.14 [26], the exact analytical estimate since it allows for less than 100% overlap with an inscribed pattern when it is tested for retrieval/recognition/recall.

## 3 Invoking orthogonalization

Our earlier papers [6, 11, 12] outline an orthogonalization scheme due to Gram Schmidt, which when combined with Hebb’s rule of learning, not only increases the memory capacity drastically but also enriches the model brain with a number of cognitively appealing features. Here we will explore the implications of orthogonalization on stability of memories, that is, how it affects the basins of attraction of the inscribed patterns.

In the orthogonalization scheme the incoming raw vectors, ***ξ*^(*ν*)^**’s, are first orthogonalized sequentially following the Gram-Schmidt procedure, then normalized and the orthonormalized vectors ${\hat{\eta }}^{\left(\nu \right)}$’s are stored in the Hebbian manner [6, 11, 12]. A significant aspect of the scheme is that although ${\hat{\eta }}^{\left(\nu \right)}$’s are stored, the network dynamics allows the raw vectors ***ξ*^(*ν*)^**’s to be retrieved or recognized in just a couple of iterations. They can even be recalled quite efficiently if patterns similar to {***ξ*^(*ν*)^**}’s are presented for association.

Our calculations show that retrieval/recognition happens for all (*N* − 1) patterns in a system of *N* neurons. This is true of both ${\hat{\eta }}^{\left(\nu \right)}$’s and ***ξ*^(*ν*)^**’s. However, the results for recall require some special discussion, which we will come to after making the general observations.

### 3.1 Basins of attraction after orthogonalization

Since the existence of basins of attraction around an inscribed pattern has been argued to be central to the criterion for stability it is necessary that we study the status of basins of attraction after orthogonalization. For this purpose we will compute the radii of basins of attraction following orthogonalization to study the effects of noise elimination on basin size.

The basins of attraction of some of the memorized patterns, ***ξ*^(*ν*)^**’s, are shown in Table 3. The basins are initially fairly large and somewhat isotropic. With increasing load, the basins shrink in size but still remain more or less uniform. This is in contrast to the scenario in the Hopfield network, where some patterns may have anisotropic basins or even lack basins even at *p* as low as 0.1*N*.

**Table 3:** Table showing the basins of attraction of some of the raw patterns, *ξ*^(*ν*)^’s, for different values of *p* for *N* = 100 after invoking orthogonalization. The basins are fairly large and uniform.

	***ξ*^(*ν*)^**	Hamming distances forming the basin of attraction of ***ξ*^(*ν*)^**									
*p* = 10	***ξ*^(1)^**	38	38	35	33	36	42	44	43	34	42
	***ξ*^(5)^**	40	36	39	42	43	43	37	44	41	41
	***ξ*^(10)^**	37	43	34	41	36	37	41	37	42	40
*p* = 20	***ξ*^(1)^**	32	32	29	34	29	34	31	29	36	37
	***ξ*^(10)^**	35	32	32	31	40	30	33	35	33	39
	***ξ*^(20)^**	35	36	34	32	39	33	28	34	26	31
*p* = 30	***ξ*^(1)^**	28	28	28	28	28	26	32	27	29	28
	***ξ*^(15)^**	25	28	29	26	29	31	21	24	20	27
	***ξ*^(30)^**	26	30	29	25	22	32	30	28	28	27
*p* = 40	***ξ*^(1)^**	23	24	17	19	20	18	19	19	17	16
	***ξ*^(20)^**	20	23	20	23	17	16	20	22	20	21
	***ξ*^(40)^**	22	17	19	19	18	17	19	19	18	18
*p* = 50	***ξ*^(1)^**	8	6	10	9	8	7	11	10	8	8
	***ξ*^(25)^**	8	8	9	11	11	7	10	9	10	12
	***ξ*^(50)^**	11	6	12	9	7	8	8	7	12	11
*p* = 60	***ξ*^(1)^**	4	3	5	6	3	3	4	5	4	7
	***ξ*^(30)^**	6	5	4	4	4	3	5	3	3	6
	***ξ*^(60)^**	5	5	4	5	4	3	4	4	3	3

### 3.2 Energy landscape and network dynamics in the modified model

We find that {***ξ*^(*ν*)^**} as well as $\left\{\hat{\eta }\right\}$ form energy minima and can all be recognized by the network, but in this current study, we focus on the stability of raw patterns. Quite remarkably, there is no shifting of minima. This is apparently because the soft noise is completely eliminated due to orthogonalization.

**Fig 3:**
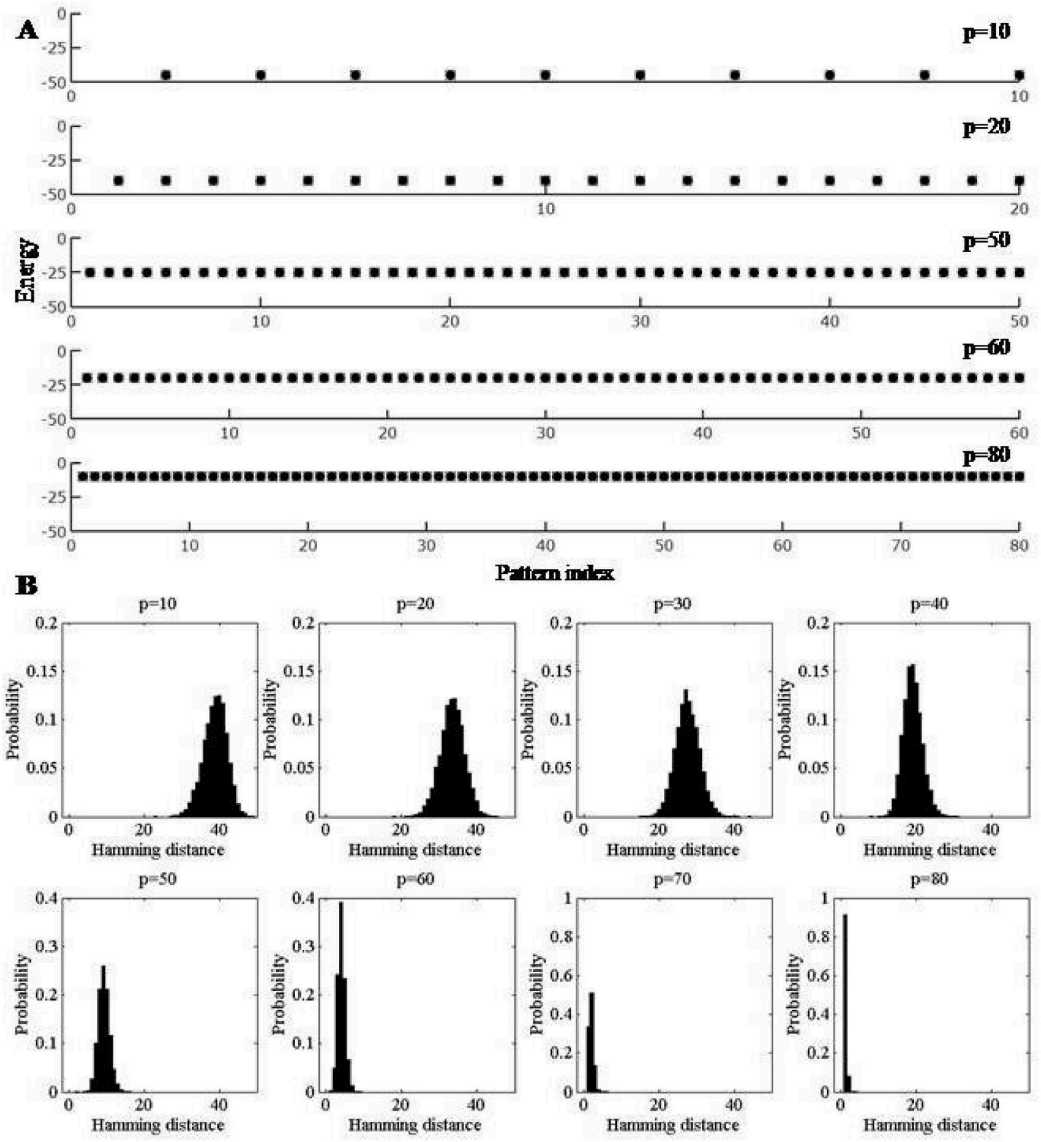
Energies and histograms characterising basins of attraction for increasing inscription of orthogonalized vectors. Fig. **(A)** shows the energy of the ***ξ***’s (■) and the attractors or energy minima (▫) for various values of *p* following the orthogonalization scheme for *N* = 100. The x-axis gives the pattern index, or pattern number for each value of *p*. As the ***ξ***’s all form energy minima, the ■ and ▫ coincide, indicating that there are no instances of minima shifting with increasing *p*. As *p* increases, the energy of the ***ξ***’s go up, but they remain as energy minima, i.e., with rising *p*, the valleys in the energy landscape shrink in size and the bottoms are at higher energy. Fig. **(B)** illustrates the evolution of basins of attraction after orthogonalization. The histograms show the distribution of the Hamming distances in the basins of attraction for different values of *p* after orthogonalization for 50 trials with *N* = 100. Each Hamming distance pertains to one particular sample of a pattern from a particular trial. The x-axis gives the range of values that Hamming distances can take, while the y-axis shows the probability for each of these values. The probability (*P*) is calculated as *P* = *f*(*x*)/*c*, where *f*(*x*) gives the number of Hamming distances with value *x*, and *c* is the total number of Hamming distances. The value of *c* is given by p*s*T, where *p* gives the number of patterns, *s* is the number of samples and *T* gives the number of trials. Note that the basins are initially large and relatively isotropic. Zeroes begin to appear around *p* = 60 and by *p* = 80, the basins of attraction are dominated by 0’s.

With the weights now being calculated using the $\left\{\hat{\eta }\right\}$, using Eq (9), we can calculate the the energy of the pattern ***ξ*^(*μ*)^** after orthogonalization as:

$$\begin{array}{c} E(\xi^{\left(\mu \right)})=-\frac{N}{2}+\frac{N}{2}(O(\frac{p}{N})), \end{array}$$ (12)

and depends on the value of *p*. As more patterns are inscribed in the network, their energies rise, as illustrated in Fig 3**(A)**. Unlike in the Hopfield model, we find that the energy of a pattern is proportional to the number of patterns in the memory store, *p*. Fig 3**(B)** illustrates the distribution of sizes of the basins of attraction of the ***ξ***’s after orthogonalization for the corresponding values of *p*. For different values of *p*, the radius of the basin of each pattern is calculated. The entire set of values is then binned, with 0 and *N*/2 specifying the minimum and maximum values a basin radius can take. The histograms show the likelihood of basin radii for each value within this range. The radii of the basins shrink with the rise in energies as memory load increases. We have seen that all the inscribed patterns always form energy minima, unlike in the Hopfield model.

**Fig 4:**
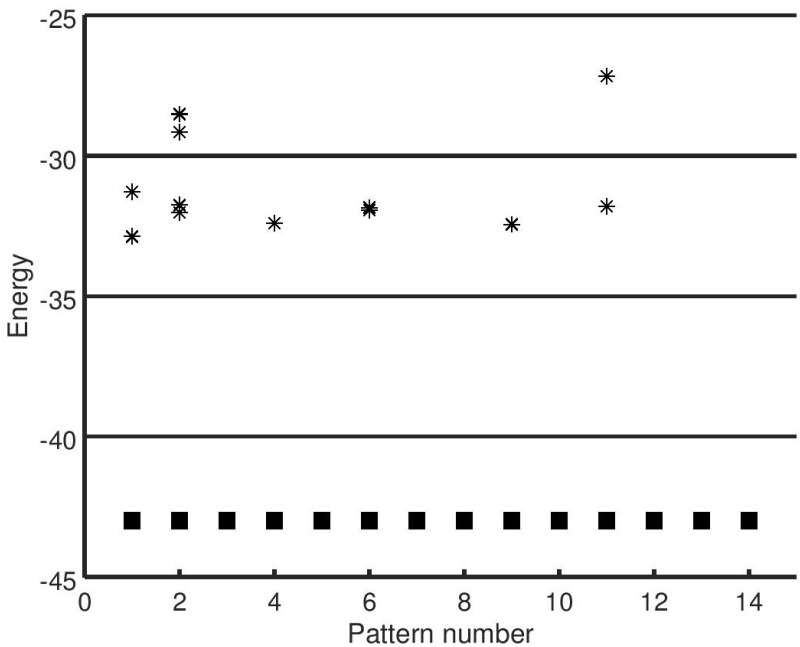
Energy of attractors (*p* = 14). Plot showing the energy (from Eq (12)) of the raw patterns, ***ξ***’s (■) and the corresponding attractors (▫) for *p* = 14 following the orthogonalization scheme. ■ and ▫ overlap each other completely for all the patterns. Some mixture states (formed from combinations of ***ξ***’s, denoted by (*’s)) are also shown for *p* = 14, plotted against the pattern ***ξ*** with which they have maximum overlap. The mixture states shown here lack basins of attraction of their own and typically converge to one of their component patterns. Refer to Fig 2**(A)** for comparison.

For completeness, we now check whether spurious minima exist for the raw vectors, ***ξ***’s, even after inscribing their orthogonalized versions. We find that the inverses of the ***ξ***’s also form minima. We also verify whether mixture states formed from combinations of ***ξ***’s form minima. At low values of *p*, mixture states are present, as shown in Fig 4, but they converge to one of the component states. At higher values of *p*, say around *p* = 50, the mixture states do not converge at all. This is to be expected, as the ***ξ***’s themselves have very small basins of attraction at high values of *p*.

### 3.3 Improved memory capacity

We have found that after orthogonalization the ***ξ***’s will all be energy minima for upto *p* = *N* − 1, i.e. the learnt patterns are all recognized for all *p* ≤ *N* − 1.

The value of the stabilization parameter will also be positive, as the noise term is now eliminated completely. But as *p* approaches *N* − 1, although $s^{\left(\nu \right)}>0$, its magnitude decreases, and so the energy of the patterns goes up and the radii of the basins of attraction also grow smaller. Eventually, a limit (*p* = 0.63*N*) is reached where some patterns may get zero basins of attraction. This limit marks the capacity of the network for associative recall. The number of patterns with zero basins of attraction increases with further memorization, but recognition remains unaffected.

**Fig 5:**
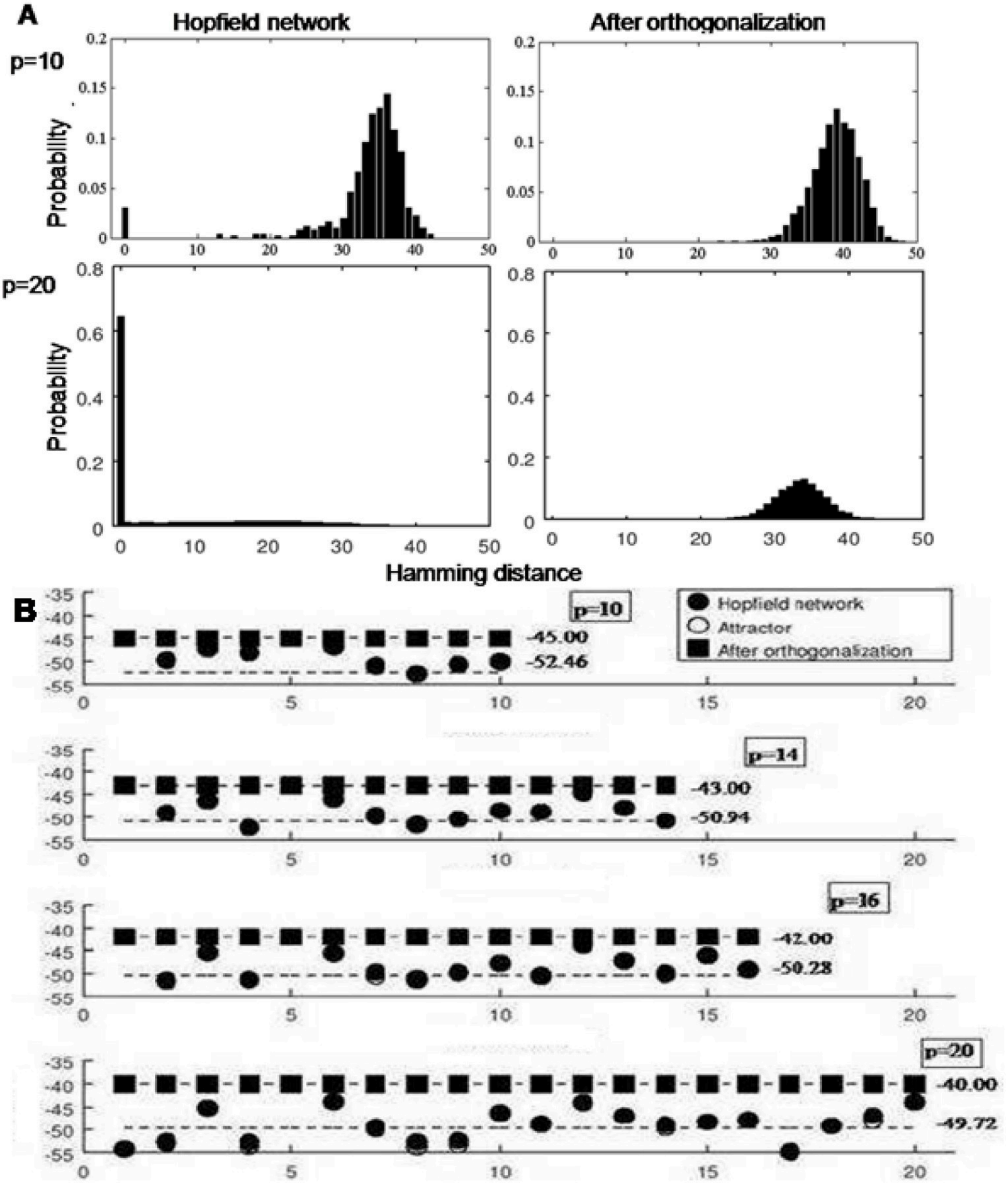
Distribution of basins of attraction before and after orthogonalization. The histograms in Fig. **(A)** show the distribution of the Hamming distances in the basins of attraction for *p* = 10 and *p* = 20 in the Hopfield network and in the network after orthogonalization for 50 trials. When *p* = 10, some of the basins of attraction in the Hopfield network become zeroes, by *p* = 20, the basins of attraction are predominantly 0. By contrast, after orthogonalization, the sizes of the basins are larger and the distribution of the Hamming distances is also relatively uniform. Fig. **(B)** plots the energy of the ***ξ***’s and the corresponding attractors ***ξ***′’s for various values of *p* (*p* = 10, 14, 16, 20) for both the Hopfield network and orthogonalization scheme. In the Hopfield network, as *p* increases, some of the ***ξ***’s are no longer minima. However, the energies of the attractors remains distributed around a mean (close to 0.5*N*). The average energy of the inscribed patterns is shown next to each value of *p*. After orthogonalization, all the ***ξ***’s have uniform energy for a particular value of *p*, and while the energy increase with the number of patterns, the ***ξ***’s remain minima.

In the Hopfield network, the basins are large and isotropic but only at very low levels of loading. One way of improving the quality of memory as an associative memory is to modify the learning rule, such as in ref. [27]. In our scheme, the process of orthogonalization endows the preferred qualities of isotropy and largeness of size on the basins of attraction. This improvement in the effectiveness of the network as associative memory is a byproduct of orthogonalization: we did not modify the learning rule to make the basins larger or more uniform, nor was there any preprocessing of input patterns involved. Fig 5 provides a comparison of the basins of attraction and energies of attractors in the Hopfield network and the orthogonalization scheme.

We should examine the implications of the changes in basin radii after orthogonalization on the memory capacity of the network and its effectiveness or quality as an associative memory. As we have understood, for a network to function well as an associative memory, the basins of attraction should preferably be large as well as isotropic [27]. Large basins would imply that each pattern has a large number of patterns associated with it, so, if the basins are vast, patterns are more likely to converge to one of the inscribed patterns than to a spurious state. Moreover, larger basin radii would mark a high level of error tolerance, as even those patterns that are some distance away from an inscribed pattern would be associated with it. Uniform or isotropic basins reduce the probability of misclassification, thereby increasing the accuracy of predictability—we can predict from the Hamming distance the inscribed pattern a random test pattern would be associated with. When the basins of attraction of all the inscribed patterns are large and fall within the same range of size, the network is unbiased towards all the patterns.

## 4 Effects of correlations in the patterns—before and after orthogonalization

Though the correlations, which are detrimental to stability of the memory in the Hopfield model, are eliminated by orthogonalization, it should be interesting to check how they would be interesting to check how they would manifest if they are reintroduced after storing the orthogonalized information. We examine the extreme case of very high similarity between the inscribed patterns in both the Hopfield network and the orthogonalization scheme. We demonstrate that even highly similar patterns can be discriminated from each other in the orthogonalization scheme. In the Hopfield network, patterns similar to a learnt (or raw) pattern tend to fall within its basin of attraction, but after orthogonalization, if two very similar patterns are learnt by the network, they are no longer associated with each other, though there may be other patterns similar to, and hence associated with, each of them. In cognitive terms, each of the attractors corresponding to the ***ξ***’s is a unique category with different items associated with it. The network is able to identify and distinguish between all the raw patterns ***ξ***’s no matter how high the degree of similarity between them exists.

In the Hopfield model a high degree of similarity between the inscribed patterns disrupts pattern stability. When two very similar patterns (with say 2 differences) are inscribed in the Hopfield network, one or both the similar patterns lose their basins of attraction: similar patterns fall within the same category, in cognitive terms. One of the two similar patterns either converges to the other, or they both converge to different patterns which are very similar to them (say to the extent of 99%), but not the same as any of the other inscribed patterns, as illustrated in Table 4. Thus one or both the patterns become unstable.

**Table 4:** Table showing examples of where patterns may converge in multiple trials in the Hopfield network and in the network after Gram-Schmidt orthogonalization when two very similar patterns are inscribed in a network of size *N* = 100. We first inscribe 4 patterns (***ξ*^(1)^** to ***ξ*^(4)^**) in the network, then choose ***ξ*^(*ν*)^** randomly from one of these 4 to make a fifth pattern ***ξ*^(5)^** which is similar (98% similarity) to it, and differs on the sites marked as ‘Differences’. We now store this fifth pattern and check whether all the 5 patterns are attractors. The inscribed patterns other than the chosen ***ξ*^(*ν*)^** remain attractors even as *p* changes from 4 to 5. We are interested in the situation with the two similar patterns, and whether ***ξ*^(*ν*)^** and ***ξ*^(5)^** are attractors (✓) or not (×), when *p* = 5. When ***ξ*^(5)^** is presented to the network, three possibilities arise: (i) ***ξ*^(5)^** falls within the basin of attraction of ***ξ*^(*ν*)^** and converges there (Case 1), (ii) ***ξ*^(5)^** is an attractor, and ***ξ*^(*ν*)^** which was previously stable now falls within the basin of attraction of ***ξ*^(5)^** (Case 2), or (iii) both ***ξ*^(5)^** and ***ξ*^(*ν*)^** converge to a third pattern which is not any of the inscribed patterns and has 99% overlap with both ***ξ*^(5)^** and ***ξ*^(*ν*)^** (Case 3). We have used examples from different trials to illustrate each of these cases.

	*ξ*^(*ν*)^ ≡ *ξ*^(5)^ (98% similarity) (ν = 1−4, chosen randomly)			Status of *ξ*^(*ν*)^ and *ξ*^(5)^ when *p* = 5					
				Hopfield network			After orthogonalization		
	Case	*ξ*^(*ν*)^	Differnces	*ξ*^(*ν*)^	*ξ*^(5)^	Remarks	*ξ*^(*ν*)^	*ξ*^(5)^	Remarks
Randomly generated patterns ***ξ***^(*μ*)^, *μ* = 1−4, (***ξ*^(1)^**—***ξ*^(4)^)**	1	***ξ*^(1)^**	56,71	✓	×	***ξ*^(1)^** is an attractor, ***ξ*^(5)^** falls within its basin of attraction.	✓	✓	***ξ*^(1)^** and ***ξ*^(5)^** are both attractors
	2	***ξ*^(4)^**	15,62	×	✓	***ξ*^(5)^** is an attractor, ***ξ*^(4)^** falls within its basin of attraction.	✓	✓	***ξ*^(4)^** and ***ξ*^(5)^** are both attractors
	3	***ξ*^(2)^**	52,91	×	×	***ξ*^(2)^** and ***ξ*^(5)^** both fall within the basin of a third attractor distict from both but 99% similar to each.	✓	✓	***ξ*^(2)^** and ***ξ*^(5)^** are both attractors

The high correlations between the patterns adds to the slow noise due to the overlap between the inscribed patterns even at loads as low as *p* = 5.

On the contrary, after orthogonalization, the high correlation between very similar patterns does not make them unstable, it only affects the sizes and shapes of their basins of attraction. When two extremely similar patterns (98% similarity, *p* = 5) are inscribed in the network after orthogonalization, they both form attractors and are recognizable. However, the extreme similarity makes the basins of attraction anisotropic where they extend to only short distances in some directions, as illustrated in Table 5.

**Table 5:** Basins of attraction for p = 4 and p = 5, with *ξ*^(5)^ (98%) similar to *ξ*^(2)^. Following the addition of a pattern very similar to one of the previously inscribed patterns, the basin of attraction of ***ξ***^(5)^ in the Hopfield network(HN) vanishes or becomes 0. After orthogonalization (GS), the basins of attraction are all non-zero, however, the addition of a similar pattern makes the basins anisotropic.

	***ξ*^(*ν*)^**		Hamming distances in the basins of attraction									
*p* = 4	***ξ*^(1)^**	HN	46	34	39	41	47	40	46	43	38	46
		GS	42	40	46	41	38	38	32	45	40	43
	***ξ*^(2)^**	HN	44	41	40	41	47	45	39	45	28	44
		GS	42	41	43	34	37	44	40	39	38	44
	***ξ*^(3)^**	HN	39	44	46	37	34	45	39	28	45	48
		GS	47	47	43	41	35	31	45	45	43	37
	***ξ*^(4)^**	HN	46	35	41	27	42	40	43	43	33	44
		GS	39	41	43	44	41	38	40	46	39	37
*p* = 5	***ξ*^(1)^**	HN	38	20	41	39	15	38	43	42	25	42
		GS	15	34	38	27	5	45	27	38	34	14
	***ξ*^(2)^**	HN	48	47	42	44	50	48	47	46	47	50
		GS	8	29	13	25	39	6	16	34	5	38
	***ξ*^(3)^**	HN	31	37	29	43	15	17	43	25	16	31
		GS	41	47	45	44	43	42	45	44	46	33
	***ξ*^(4)^**	HN	40	40	38	34	34	40	26	44	38	33
		GS	4	41	38	42	18	4	39	2	43	30
	***ξ*^(5)^**	HN	0	0	0	0	0	0	0	0	0	0
		GS	38	30	7	16	16	30	41	44	44	42

While we have shown here an example of the effects of correlations for low memory loads, we find computationally that the above observations are still valid at higher values of *p* (*p* = 10, 20, 30…, 90). After orthogonalization, the presence of even a single pair of very highly correlated patterns affects the radii of the basins of attraction and also introduces anisotropy when the basins are non-zero. Recognition remains unaffected even in the presence of highly correlated patterns. By contrast, a high degree of correlations is destructive to both recognition and recall in the Hopfield network, as the two processes are interlinked– recognition guarantees recall, as the attractors are always surrounded by finite-sized basins while recall is present only when there is recognition, as attractors are (first and foremost) stable fixed points, by their very nature.

The above analysis leads to the following important result. While, as we have discussed above, in the Hopfield model, as *p* increases the inscribed patterns increasingly lose their attractor nature, and so their stability as memories, invocation of orthogonalization allows all the inscribed patterns to retain their stability as memories in that they are always *retrieved* or *recognized*; however, *recollection* of an inscribed pattern from an inexact input patterns acquired a novel status for *p*/*N* close to 1. Quite understandably the basins of attraction shrink with increasing *p* while the inscribed patterns remain pinned at the minima, beyond *p* ≈ 0.63*N* some of the basins reduce in size making them look like inverted *δ*−functions, i.e. there is a single recognizable pattern per basin.

Such inscribed patterns do not have another learnt pattern, however close to them, that can be attracted to them. Since a basin of attraction is viewed as something enclosing a category of patterns similar to the inscribed one that lies at the bottom, the single pattern inverted *δ*−function like basins represent unique and extreme situations in which each pattern is a category by itself, i.e. specifications ought to be given to the last detail to identify an entity/object. We often come across such situations in real life. Here we see how orthogonalization can help us realize them.

## 5 Method

The network was built on GNU Octave/MATLAB. The size of the network was fixed at *N* = 100. The patterns were randomly-generated *N*-dimensional vectors with components having different signs but the same magnitude, namely 1. The patterns were stored using the learning rule of Eq (1) and tested for retrieval and recognition and recall following Eq (3). The data was obtained always from 50 sets of patterns (or trials), collated and then arranged into bins. Recall of a learnt pattern was tested by calculating its basin of attraction—in a learnt pattern chosen to test for recall the signs of its components, visualized as spins, are flipped systematically in a random sequence (which is called a sample) and convergence is checked at each step; the process is stopped when there is no more convergence. The number of flipped spins gives the Hamming distance for that sample. The process is repeated with different samples and the set of all Hamming distances gives the basin of attraction for that pattern. This protocol is described in greater detail in the box in the S1 Appendix.

## 6 Discussion

The orthogonalization scheme leads to an increase in the memory capacity of the network. The capacity for recognition goes up from *p* = 0.1*N* in the Hopfield network to *p* = *N* − 1, as the ***ξ***’s are all energy minima as long as *p* < *N* − 1. The capacity for associative recall is now around 0.63*N* and within this limit, the ***ξ***’s all have non-zero basins of attraction. Unlike in the Hopfield network where recognition and recall are interdependent, after orthogonalization, recognition does not necessarily indicate the possibility of recall.

Further, as we have seen with orthogonalization, the condition 1 for pattern stability (from sec. 2.2), namely recognition, is met in spite of the condition 2, pertaining to recall, not being satisfied. This prompted us to identify the first two conditions as a necessary condition (implying recognition) and a sufficient one (meaning recall).

Another important implication of orthogonalization is that in our model, the processes of *pattern separation* and *pattern completion* can be separated from each other. Pattern separation, or isolating individual patterns regardless of the degree of correlations between them, is essentially the process of orthogonalization. Pattern completion is simply the associative recall of a correct inscribed pattern on presenting an erroneous or partial version of the pattern to the network. This dissociation of the two processes has also been implicated in biology with different parts of the hippocampus believed to be responsible for each [15, 16].

We have discussed how energy landscape changes dynamically with increasing *p*. Almost every aspect of it is cognitively relevant. A basin of attraction is like a category of similar entities labeled by the attractor pattern at the bottom of the basin. It is natural that the categories should get reorganized and refined as we add more information through new patterns. This is reflected in the shrinking of basins and creation of new ones as *p* increases. Addition of new information can change the perception of certain known entities. This well-known happening is manifested in certain minima (belonging to inscribed patterns) transforming into new minima that do not correspond to known inscribed patterns.

What does not appear to have a cognitive analogue is failure of recognition or recall for very large values of *p* in the Hopfield model. This is where the role of orthogonalization comes. It provides the model brain with a robustness in retrieval/recognition/recall and in category formation.

In biological terms orthogonalization could possibly be performed in cerebellum and hippocampus, more specifically CA3, where there is evidence of attractor dynamics happening [28, 29]. Refer to [30, 31] for a model of recognition in the perirhinal cortex.

## 7 Conclusion

We have attributed precise mathematical definitions to the terms retrieval, recognition and recall, and redefined pattern stability in terms of a necessary and a sufficient condition, representing recognition and recall respectively. Having explored the energy landscape for Hopfield network in its entirety we moved to networks that stored orthogonalized versions of incoming formation. Orthogonalization brings about drastically new results for pattern stability and associative recall when it is incorporated in the framework of Hopfield model, in addition to improving the memory capacity and efficiency of the network as an associative memory. However, the catastrophic interference due to the correlations between the learnt patterns is not eliminated completely, though its effects are delayed significantly.

There are a few strategies reported in literature [32–35] for increasing the memory capacity beyond what is set by the Hopfield model. However, the advantage of the orthogonalization scheme over the above mentioned strategies to overcome catastrophic breakdown of memory happening in Hopfield network is that it does not require any drastic changes to either the network topology or the learning rule.

Experiments are needed to understand the exact biological correspondence of the dynamics of our model. Ref. [36] and models based on *in vivo* data, such as [34, 35] and [37] can provide some insights into the changes in dynamics of Hopfield network on introduction of orthogonalization. Note that in reality memories are not supposed to be stable. Each time a memory is recovered it has to be reconsolidated by lodging it back in the brain all over again [38, 39]. In this process the memory becomes labile and can be stored in an altered form. However, the notion of stability as studied in this paper is *not* in conflict with the notion of lability of memories. Our concern is the robustness (or stability) of memories against the incessant piling up of information in the synapses. Thus there is a clear need for a generalization of the present work to accommodate the labile nature of memories by treating the problem as an interplay of stability and flexibility.

It would be interesting to extend the results obtained here to other orthogonalization schemes [40, 41], and to examine the cognitive relevance of various orthogonalization schemes and compare them. The network dynamics after orthogonalization in the case of networks with sparse coding should also be important to study.

## Supporting information

S1 Text Spurious minima in the energy landscape. [7, 10]. (PDF)

S1 Appendix (PDF)
