# Supplementary material for: On stability and associative recall of memories in attractor neural networks
Source: PLoS One. 2020 Sep 17;15(9):e0238054. doi: 10.1371/journal.pone.0238054 (PMC7498056; doi:10.1371/journal.pone.0238054)
Supplement: S1 Text — (PDF) [file pone.0238054.s001.pdf]

S1 Text. Spurious minima in the energy landscape. The mirror images of the inscribed patterns are also attractors in the energy landscape and have basins of attraction similar to those of the corresponding inscribed patterns, as seen in S1 Table. The configuration space can thus be considered to consist of two halves, one half containing the attractors due to the inscribed patterns and the other, their inverses.

Apart from the inscribed patterns and their inverses, there are other forms of attractors in the energy landscape. These *spurious minima* are typically formed from combinations of the inscribed patterns. Such states formed by the combination of an odd number of patterns are stable fixed points, while the even combinations make saddle points [7]. These mixture states are referred to as *symmetric* mixtures as they have roughly similar overlaps with their component states. In addition to these, there are other *asymmetric* mixture states which also form minima. The asymmetric mixtures are not simple combinations of the component states, but involve prefactors which can be integers as well as fractions. For instance, $\boldsymbol{\xi}^{\boldsymbol{(mix)}} = sgn\left(\boldsymbol{\xi}^{\boldsymbol{(1)}} + \boldsymbol{\xi}^{\boldsymbol{(12)}} + \boldsymbol{\xi}^{\boldsymbol{(14)}}\right)$ may be a symmetric mixture state for $p = 14$, while
$\boldsymbol{\xi}^{\boldsymbol{(mix)}} = sgn\left(2\boldsymbol{\xi}^{\boldsymbol{(10)}} + (3/8)\boldsymbol{\xi}^{\boldsymbol{(2)}} + (2/5)\boldsymbol{\xi}^{\boldsymbol{(4)}}\right)$ constitutes an asymmetric mixture.

These spurious minima have finite overlaps with all the inscribed patterns, but much higher overlaps with their components. They are termed as spurious because they have much higher energy than those of the stable inscribed patterns, as shown in Fig. 2(**A**). Consequently, addition of a small amount of noise (by raising the temperature, say by flipping the spins) can bring a pattern out of the basins of attraction of such a spurious attractor.

These mixture states may fall anywhere within the basin of attraction of one of the inscribed patterns or their inverses. They may also lie in the region between the basins of attraction of the stored patterns. The basins of such spurious states are generally very small in radius, as shown in S2 Table. These spurious attractors can thus be considered to be pseudoattractors or local and shallow minima with high energy. The number of such spurious patterns increases combinatorially with p. This increase causes basins of attraction of the inscribed patterns to shrink, as these spurious minima also occupy considerable area within the configuration space. In fact, the landscape is inundated with shallow and small basins due to spurious states, which restrict the space for the basins for attractors, and also cause them to displace from their original positions. In the limiting situation when none of the inscribed patterns has a basin of its own, the memory exhausts its capacity and the network enters what is called a spin glass state Ref. [7, 10] in which the synaptic weights become more or less random in the sense that they cannot connect a given inscribed pattern with itself following eq. (3) and eq. (4).

S1 **Table. Table showing the basins of attraction of some inscribed patterns ($\boldsymbol{\xi}^{(\nu)}$'s) and their inverses($\boldsymbol{\xi}_{inv}^{(\nu)}$'s) for different values of p for $N = 100$. For stable patterns, the basins of attraction of the inverse or mirror states are similar to those of the inscribed patterns. The inverses of the unstable states (with no basins) may have basins of attraction.**

	$\boldsymbol{\xi}^{(\nu)}$	Hamming distances in the basin of attraction of $\boldsymbol{\xi}^{(\nu)}$									
$p = 10$	$\boldsymbol{\xi}^{(1)}$	31	29	37	35	40	24	30	34	41	31
	$\boldsymbol{\xi}_{inv}^{(1)}$	39	32	40	33	40	34	42	38	43	35
$p = 12$	$\boldsymbol{\xi}^{(1)}$	32	36	32	44	34	34	35	32	34	37
	$\boldsymbol{\xi}_{inv}^{(1)}$	32	35	30	35	42	40	27	32	39	36
	$\boldsymbol{\xi}^{(7)}$	0	0	0	0	0	0	0	0	0	0
	$\boldsymbol{\xi}_{inv}^{(7)}$	13	6	0	0	12	36	0	19	0	14
$p = 14$	$\boldsymbol{\xi}^{(1)}$	26	31	36	41	37	22	30	31	31	39
	$\boldsymbol{\xi}_{inv}^{(1)}$	12	26	14	15	34	41	32	18	19	20
	$\boldsymbol{\xi}^{(7)}$	0	0	0	0	0	0	0	0	0	0
	$\boldsymbol{\xi}_{inv}^{(7)}$	0	10	8	5	13	14	0	10	18	0

S2 **Table. Table showing the basins of attraction of some mixture states for $p = 14$ with $N = 100$. A mixture state $\boldsymbol{\xi}^{(mix_{m,n,q,\cdots})}$ is formed from the combination of some of the inscribed patterns, viz. its 'Components' ($\boldsymbol{\xi}^{(m)}$,$\boldsymbol{\xi}^{(n)}$,$\boldsymbol{\xi}^{(q)}$, $\cdots$) and converges to an attractor $\boldsymbol{\xi}^{(\mu)}$. This $\boldsymbol{\xi}^{(\mu)}$ can be one of the inscribed patterns, typically the component with which $\boldsymbol{\xi}^{(mix_{m,n,q,\cdots})}$ has maximum overlap, or $\boldsymbol{\xi}^{(mix_{m,n,q,\cdots})}$ itself . When $\boldsymbol{\xi}^{(mix_{m,n,q,\cdots})}$ converges to $\boldsymbol{\xi}^{(\mu)}$, it lacks a basin of attraction of its own, but when it converges to itself, the basin of attraction is very small as seen below.**

Components of $\boldsymbol{\xi}^{(mix_{m,n,q,\cdots})}$ ($\boldsymbol{\xi}^{(m)}$,$\boldsymbol{\xi}^{(n)}$,$\boldsymbol{\xi}^{(q)}$, $\cdots$)	Attractor $\boldsymbol{\xi}^{(\mu)}$	Basin of attraction of $\boldsymbol{\xi}^{(mix_{m,n,q,\cdots})}$ Hamming distances									
$\boldsymbol{\xi}^{(1)}$,$\boldsymbol{\xi}^{(3)}$,$\boldsymbol{\xi}^{(4)}$	$\boldsymbol{\xi}^{(1)}$	0	0	0	0	0	0	0	0	0	0
$\boldsymbol{\xi}^{(2)}$,$\boldsymbol{\xi}^{(5)}$,$\boldsymbol{\xi}^{(8)}$	$\boldsymbol{\xi}^{(3)}$	0	0	0	0	0	0	0	0	0	0
$\boldsymbol{\xi}^{(3)}$,$\boldsymbol{\xi}^{(6)}$,$\boldsymbol{\xi}^{(4)}$,$\boldsymbol{\xi}^{(8)}$,$\boldsymbol{\xi}^{(11)}$	$\boldsymbol{\xi}^{(mix_{3,6,4,8,11})}$	0	0	0	6	2	0	1	0	0	3
