# Supplementary material for: On stability and associative recall of memories in attractor neural networks
Source: PLoS One. 2020 Sep 17;15(9):e0238054. doi: 10.1371/journal.pone.0238054 (PMC7498056; doi:10.1371/journal.pone.0238054)
Supplement: S1 Appendix — (PDF) [file pone.0238054.s002.pdf]

S1 Appendix

Calculating the basin of attraction of a pattern

Step 1: Consider test pattern $X = \boldsymbol{\xi}^{(\boldsymbol{\nu})}$. Present X for retrieval following eq. (3) to obtain X'. If $X' = X$, then X' has converged to X.

Step 2: Generate a random sequence of $N/2$ elements($S1$) for flipping, referred to as a sample. For example, for $N = 10$,

S1	10	7	5	3	6

Obtain pattern X_1 by flipping the spin of X corresponding to the first element of the sequence. Present X_1 for retrieval to obtain X_1'. If $X_1' \neq X_1$, then present X_1' for retrieval to obtain $X_1^{(1)}$. The 1 in the superscript denotes an *iteration*. Fix the maximum number of iterations (at 10, say).

X	1	-1	-1	-1	1	-1	1	-1	*1*
X_1	1	-1	-1	-1	1	-1	1	-1	*-1*
X_1'	1	-1	-1	-1	1	-1	1	-1	*1*
$X_1^{(1)}$	1	-1	-1	-1	1	-1	1	-1	*1*

Suppose $X_1^{(1)} = X_1'$, then we can say that X_1' has converged. If $X_1^{(1)} = X$, then we can say that X_1 converges to X in one iteration. That is, pattern X with one flip is associated with pattern X.
(If X is an attractor, then $X_1' = X_1$ would not be possible, as convergence happens only at an attractor.)

Step 3: Obtain pattern X_2 by flipping two elements in X following sequence $S1$. Present X_2 for retrieval and check for convergence.

X	1	-1	-1	-1	1	*-1*	1	-1	*1*
X_2	1	-1	-1	-1	1	*1*	1	-1	*-1*
$X_2^{(1)}$	*-1*	*1*	-1	-1	1	-1	1	-1	*-1*
$X_2^{(2)}$	1	-1	*1*	-1	1	*1*	1	-1	*1*
$X_2^{(3)}$	1	-1	-1	-1	1	*-1*	1	-1	*1*
$X_2^{(4)}$	1	-1	-1	-1	1	*-1*	1	-1	*1*

In this case, we can say that X_2 converges to X in 3 iterations. Then, X with 2 flips is also associated with X.

Step 4: Obtain pattern X_b with b flips. If it converges to X, then present X_{b+1} with $b+1$ flips. If there is no convergence to X even after 10 iterations, then stop the process.

Step 5: The maximum number of flips b such that X_b converges to X within a fixed number of iterations (here, 10) gives b_1, the Hamming distance for sample $S1$ for pattern $\boldsymbol{\xi}^{(\boldsymbol{\nu})}$.

Step 6: Generate another random sequences $S2$, $S3$, ..., $S10$ and present the new pattern with elements flipped according to this sequence to obtain b_2,b_3,b_4, ..., b_{10}, the Hamming distance for samples $S2$, $S3$, ..., $S10$ for 10 samples.

Step 7: The set $B = \{b_1, b_2, \ldots, b_s\}$ gives the basin of attraction of pattern $\boldsymbol{\xi}^{(\boldsymbol{\nu})}$ for s samples.

In the case of $\boldsymbol{\eta}$'s , we start with $X = \boldsymbol{\eta}^{(\boldsymbol{\nu})}$ and for further iterations, we present X' with signs obtained from eq. (4) *(with $\eta_i^{(\nu)}$ in place of $\xi_i^{(\nu)}$) calculated for $\boldsymbol{\eta}^{(\boldsymbol{\nu})}$ and the magnitude of X.*
